# 1937. Use of Paxlovid for Treatment of Acute COVID-19 and Occurrence of Post-COVID Conditions among Children and Adults at High-Risk for Severe COVID-19, April 1 - December 31, 2022

**DOI:** 10.1093/ofid/ofad500.2468

**Published:** 2023-11-27

**Authors:** Alexandra F Dalton, Sarah Baca, Julia Raykin, Cria Perrine, Tegan Boehmer, Emilia Koumans, Priti Patel, Sharon Saydah

**Affiliations:** Centers for Disease Control and Prevention, Raleigh, NC; GAP Solutions Inc., Austin, Texas; CDC, Atlanta, Georgia; CDC, Atlanta, Georgia; Centers for Disease Control and Prevention, Raleigh, NC; CDC, Atlanta, Georgia; Centers for Disease Control and Prevention, Raleigh, NC; Centers for Disease Control and Prevention, Raleigh, NC

## Abstract

**Background:**

Data have suggested that treatment with Paxlovid for acute COVID-19 decreases the incidence of Post-COVID Conditions (PCC); however, results are limited to specific age groups and populations. This analysis assessed the occurrence of PCC following receipt of Paxlovid among children and adults at risk for severe COVID-19.

**Methods:**

Patients aged ≥ 12 years with COVID-19 (defined by ICD-10 code, positive SARS-CoV-2 test, or Paxlovid prescription) and at increased risk for severe COVID-19 due to age, underlying conditions, or immune-suppressing medications, were identified in HealthVerity claims data. Eligible persons had an outpatient, telehealth, or emergency department encounter for COVID-19 between April 1 – August 31, 2022. Exclusions included liver disease, end-stage renal disease, a prescription for contraindicated medications, pregnancy in the previous year, or death within 60 days. Case patients received a Paxlovid prescription within ±5 days of the index date; control patients matched on age, sex, month, and region did not receive Paxlovid within ±5 days. Cases and controls were matched 1:2. Analysis was conducted separately for 3 age groups: 12-17 years, 18-49 years, and ≥50 years. PCC was defined as new conditions > 60 days after index date and prior to December 31, 2022. We calculated the relative risk (RR) of two overall PCC indicators (≥ 1 or ≥ 2 conditions) and 45 individual conditions.

**Results:**

Among adults aged ≥ 50 years, the risks of overall and individual PCCs were generally lower among case- than control patients (RR for ≥1 condition=0.92, 95% CI=0.91-0.93; Figure 1). There was no overall difference in risk among adults 18-49; the RR of individual conditions varied (Figure 2). Among adolescents, the risks of overall and individual PCCs were higher among case than control patients (RR for ≥1 condition=1.07, 95% CI=1.02-1.12; Figure 3).

**Figure 1.**
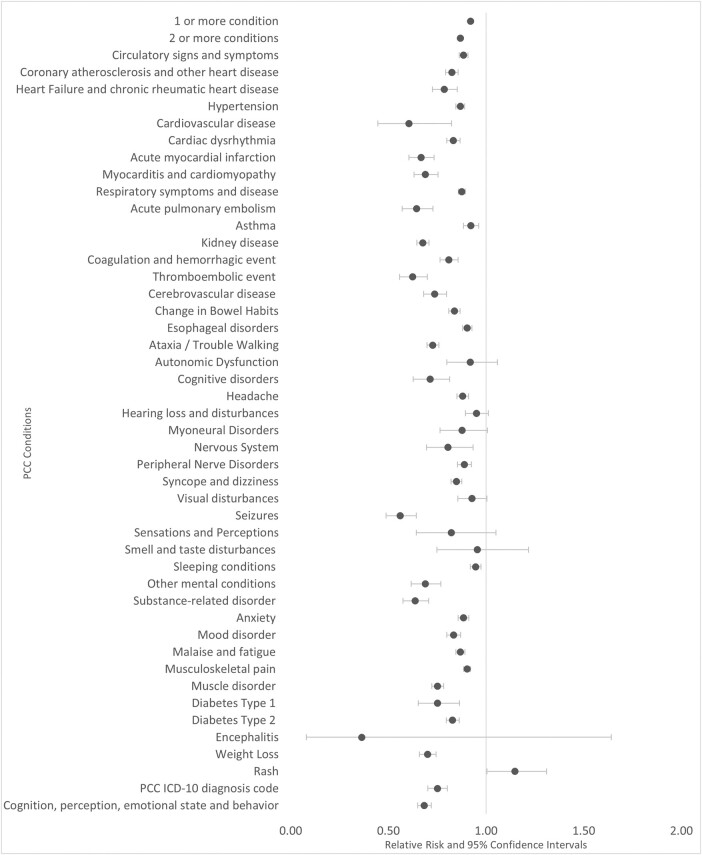
Relative Risk of Post-COVID Conditions among Patients who Received Paxlovid, Ages ≥50 (N=564,303)

**Figure 2.**
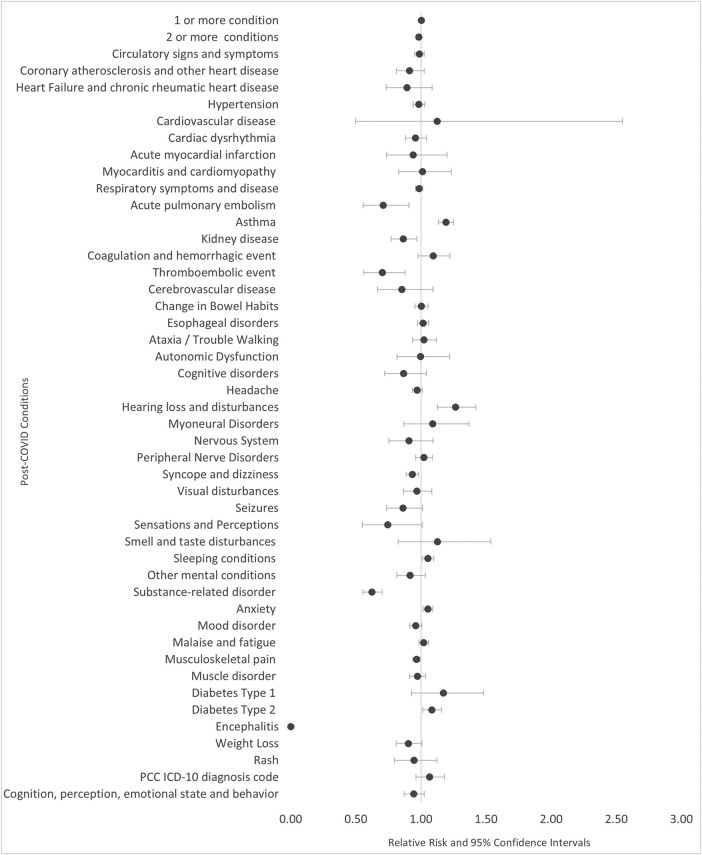
Relative Risk of Post-COVID Conditions among Patients who Received Paxlovid, Ages 18-49 (N=292,818)

**Figure 3.**
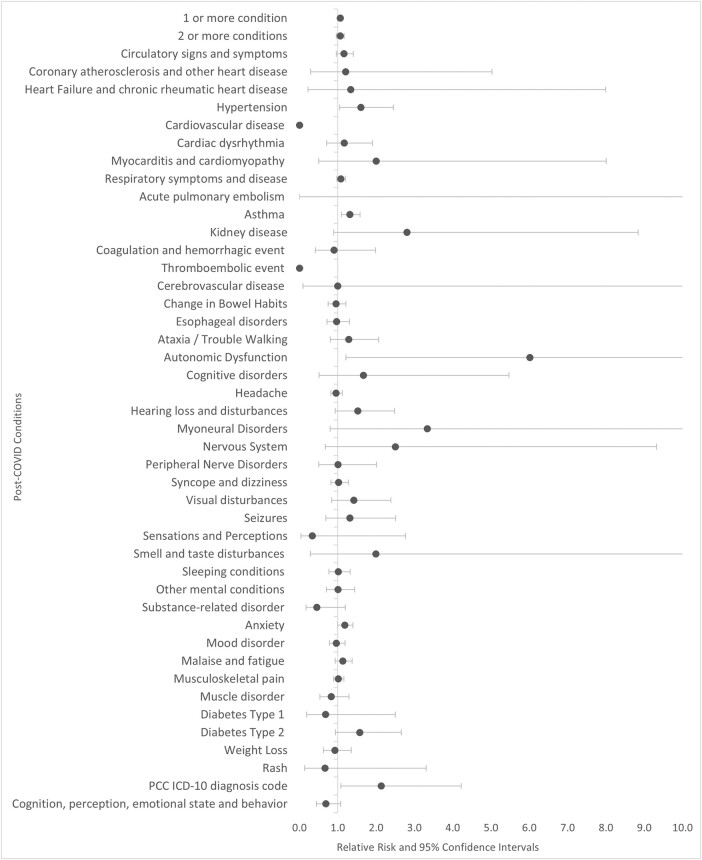
Relative Risk of Post-COVID Conditions among Patients who Received Paxlovid, Ages 12-17 (N=17,178)

**Conclusion:**

The results suggest that Paxlovid helps reduce occurrence of PCC in adults aged ≥ 50 years. Among younger adults and adolescents, associations were observed for only certain conditions. This may be due to a difference in baseline health in these age groups. Further investigation may help clarify whether Paxlovid provides benefits in reducing PCC in addition to severity of acute COVID-19.

**Disclosures:**

**Priti Patel, MD, MPH**, Pfizer: Stocks/Bonds

